# Estimating the Incidence and Prevalence of Dysphagia in New Zealand

**DOI:** 10.1007/s00455-023-10662-2

**Published:** 2024-01-20

**Authors:** Shnece Duncan, Andrea Menclova, Maggie-Lee Huckabee

**Affiliations:** 1 Department of Economics and Finance, University of Canterbury, Christchurch, New Zealand; 2 Department of Communication Disorders, Rose Centre for Stroke Recovery and Research, University of Canterbury, Christchurch, New Zealand

**Keywords:** Projected dysphagia incidence, Projected dysphagia prevalence, Dysphagia demographic composition, Dysphagia by medical condition

## Abstract

Dysphagia imposes a substantial economic burden on global healthcare systems due to its pervasive, high-cost nature. To comprehend this burden, we must first assess dysphagia's prevalence and incidence in the general population. Existing studies on dysphagia prevalence encompass minor symptoms, while it is the severe cases that drive significant healthcare costs. We address this knowledge gap by estimating dysphagia incidence and prevalence in the New Zealand population, projecting future demographics of affected individuals. Incidence and prevalence rates of dysphagia within specific underlying medical conditions are sourced from existing literature. Median projected population estimates from Statistics New Zealand, by age, sex, and ethnicity are used to calculate dysphagia projections. Where possible, projections by age and ethnicity are provided until 2038 and projections by age and sex until 2073. In 2020, 9300 New Zealanders are estimated to have newly developed dysphagia while 1.5% of the general New Zealand population are estimated to have been living with the effects of the condition. By 2073, the number of individuals newly diagnosed annually is projected to increase to 24,500 and the prevalence of dysphagia is projected to increase to 2.6%. These results indicate that a significant number of New Zealanders are impacted by dysphagia. This number is predicted to dramatically increase in the future, mostly due to population ageing, indicating an increased burden on society and healthcare systems. Our work provides a useful starting point for countries worldwide to assess future healthcare resource demands associated with dysphagia, assisting with healthcare provision planning.

## Introduction

Dysphagia presents a pervasive and intricate challenge that extends its impact across diverse populations and healthcare systems worldwide. This complex condition not only affects the quality of life for individuals but also poses significant economic burdens on healthcare systems. The burden is exacerbated by the global demographic trend of ageing populations [1], as dysphagia is notably prevalent among older individuals. As the world grapples with the profound implications of an ageing society, the prevalence of dysphagia continues to escalate, presenting an imminent and growing economic challenge to healthcare systems on a global scale. To better understand the current and future economic burden of dysphagia, we must first evaluate the prevalence and incidence of the disorder. While the prevalence of dysphagia has been well researched within its various underlying conditions, there has been little done internationally to estimate its prevalence within the general population. Rajati et al. [2] provide a meta-analysis on research investigating the prevalence of oropharyngeal dysphagia within different populations and identify three studies that focus on the general population. Prevalence rates of dysphagia within the general population of Australia, Sweden, and Argentina are reported as 7.3, 10.0, and 29.6%, respectively. These studies utilise a broad definition of dysphagia, including relatively minor symptoms of impaired swallowing such as gastroesophageal reflux disease (GERD). While these relatively minor symptoms are problematic and undesirable for individuals, it is the more serious cases of dysphagia which are economically significant, incurring higher healthcare costs. To understand the extent of the economic burden of dysphagia, estimates should focus exclusively on the prevalence of more serious symptoms of oropharyngeal and oesophageal dysphagia and exclude GERD symptoms. Just one study internationally—that we are aware of—follows this narrower definition of dysphagia in estimating the prevalence of dysphagia in the New Zealand general population [3]. However, the research does not consider either the demographic composition of the dysphagic population or the incidence rate of dysphagia. We address this critical international knowledge gap by investigating the incidence and prevalence of dysphagia within the general population in a New Zealand setting. By excluding relatively minor definitions of dysphagia from our analysis, we estimate the extent to which dysphagia presents considerable economic burden both currently, and into the future.

## Methods

Incidence and prevalence rates of medical conditions that cause dysphagia are sourced from existing literature (see Appendix Table 1). Age- and sex-specific rates are then applied to median projected population estimates from Statistics New Zealand [4, 5] to estimate the future incidence and prevalence of each medical condition up to year 2073. Where available (i.e., up to year 2038), age- and ethnicity-specific rates are sourced to analyse the future demographic composition of the patient population.^1^

The prevalence of dysphagia (oropharyngeal and oesophageal) within each underlying medical condition is then investigated. Despite focusing solely on oropharyngeal and oesophageal dysphagia, and excluding GERD symptoms, reported rates of dysphagia prevalence vary widely depending on the diagnostic method used. More sensitive diagnostic tools detect dysphagia within patients that other methods can miss. Lower and upper bounds of dysphagia prevalence enable this analysis to capture the range of estimates from existing literature (see Appendix Table 2).

## Results

### Incidence of Dysphagia

**Fig. 1:**
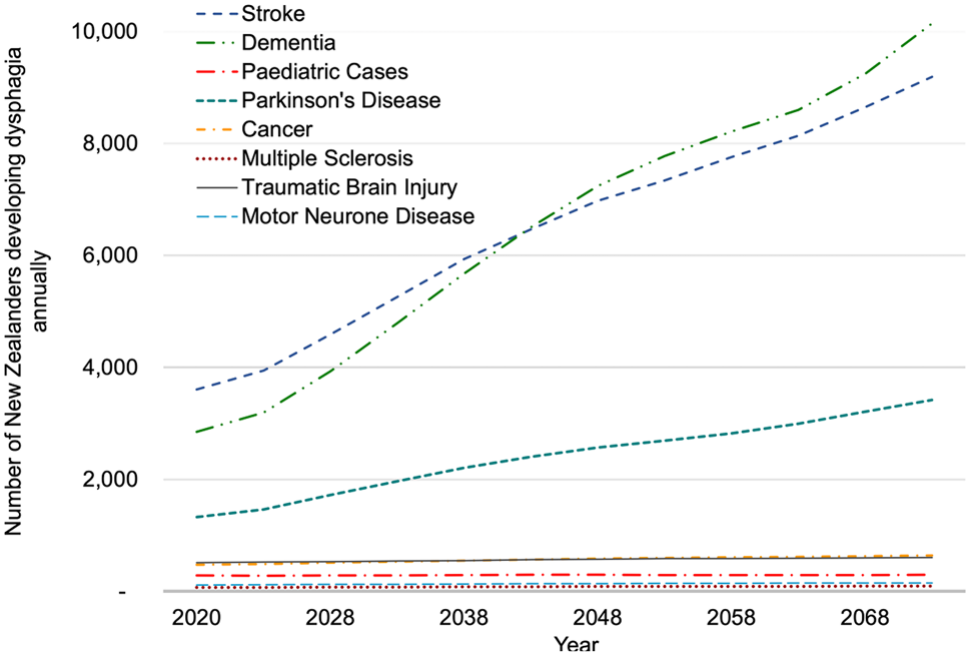
Projected Dysphagia Incidence in New Zealand by Medical Condition (2020–2073)

We project the total incidence of dysphagia in New Zealand to increase from 9,250 (7000–12,500) individuals in 2020 to 24,500 (18,700–32,850) individuals in 2073. This corresponds to an incidence rate of 182 individuals per 100,000 people in the general New Zealand population in 2020, increasing to 361 per 100,000 by 2073. Figure 1 displays our incidence projections by medical condition. We can see that stroke, dementia and Parkinson’s disease contribute a significant portion of the total incidence and are largely responsible for the increase in total incidence projected up to 2073. This is mostly due to the incidence rates of these conditions increasing with age, alongside the projected population ageing. Other conditions including paediatric cases, traumatic brain injury, cancer, motor neurone disease and multiple sclerosis contribute a smaller and relatively constant number annually.

**Fig. 2:**
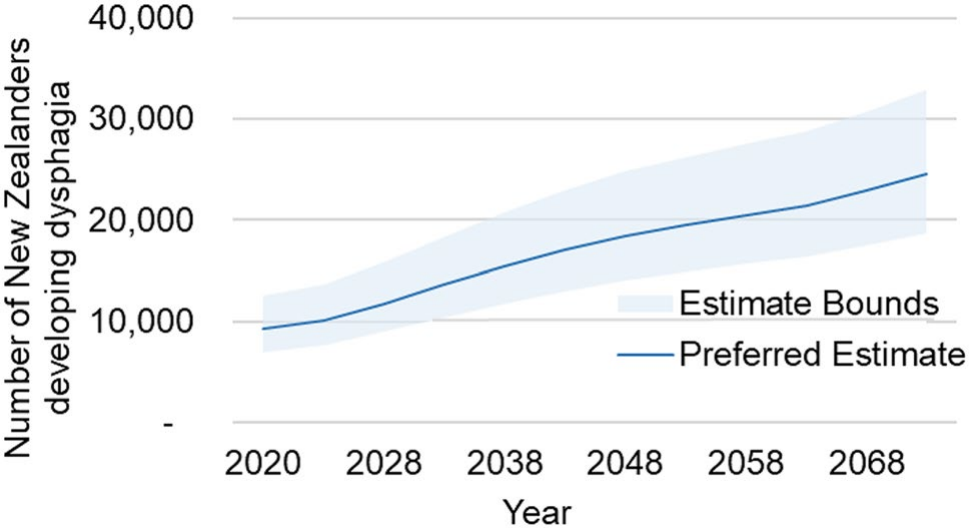
Projected Dysphagia Incidence in New Zealand 2020–2073—Lower and Upper Bounds

Lower and upper bounds for the projected total incidence are included in Fig. 2. Given that the 2020 upper bound estimate is lower than the 2073 lower bound estimate, we are confident in projecting an increasing incidence of dysphagia in New Zealand.

Of the total incidence data in 2020, 97% is sex-specific while in 2073, 99% is sex-specific. This is due to having sex-specific data available only for some underlying medical conditions. From sex-specific data, we calculate 49% of the new patients to be male and 51% female in both 2020 and 2073. It is therefore unlikely that there will be large sex disparities in new cases of dysphagia into the future. Of the total incidence data in 2020, 93% is age-specific while in 2073, 96% is age-specific. From age-specific data, we calculate 22% of the new cases in 2020 to be among individuals under 65 years old and 78% among 65 + year olds. Strikingly, by 2073, we project that individuals under 65 will only be responsible for 9% of new cases of dysphagia and those 65 + will be responsible for 91%. Therefore, of the total new cases of dysphagia expected annually, a significantly increasing proportion are likely to be in the older age cohort.

Ethnicity-specific data represents 84% of the total incidence data in 2020 and 93% in 2073. This is due to having ethnicity-specific data only for some underlying medical conditions. We calculate that from 2020 to 2073, the proportion of NZ Europeans in the total dysphagia incidence will decrease from 82 to 75%, the proportion of Māori will increase slightly from 8 to 9% and the proportion of Pasifika from 4 to 5%, while the proportion of Asians will increase significantly from 6 to 11%. It is therefore likely that most new cases of dysphagia will continue to be among NZ Europeans; however, we should expect an increased proportion of new patients of Asian ethnicity in the future.

### Prevalence of Dysphagia

**Fig. 3:**
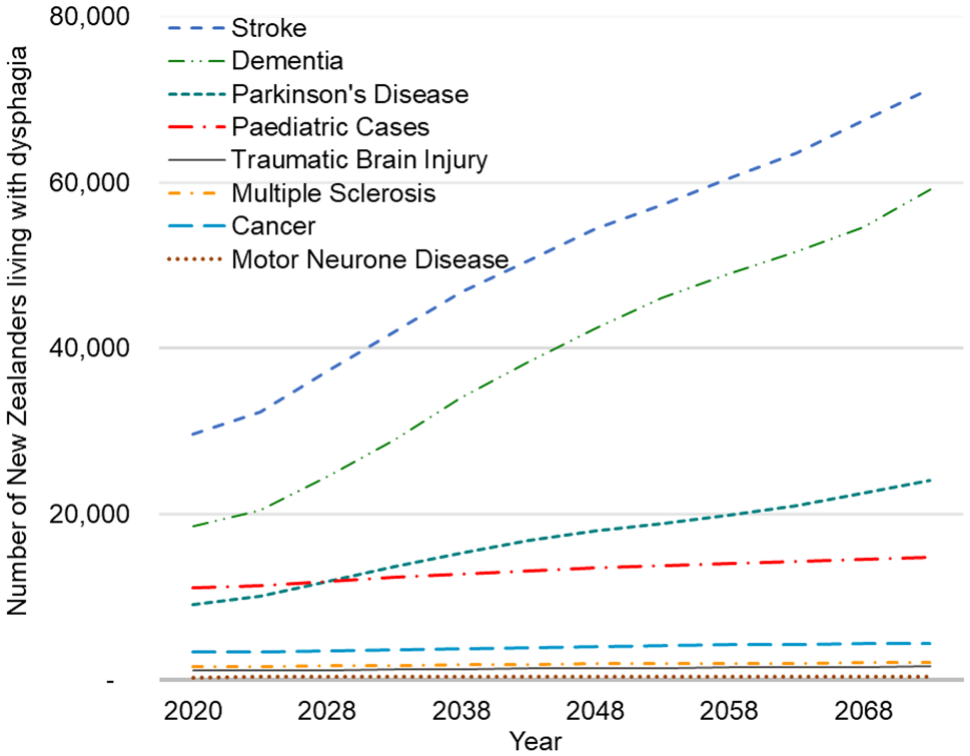
Projected Dysphagia Prevalence in New Zealand by Medical Condition (2020–2073)

We project the total prevalence of dysphagia in New Zealand to increase from 74,600 (56,000–102,100) individuals in 2020 to 177,600 (134,700–240,900) individuals in 2073. In 2020, this corresponds to a prevalence rate of 1,470 individuals per 100,000 people or 1.5% (1.1–2.0%) of the total New Zealand population. We project this to increase by 2073 to 2610 individuals per 100,000 people or 2.6% (2.0–3.5%) of the total population. Figure 3 displays the prevalence projections by medical condition. We can see that stroke, dementia, Parkinson’s disease and paediatric cases contribute to a significant portion of the total prevalence. Stroke, dementia and Parkinson’s disease also contribute significantly to the increase in total prevalence projected by 2073. This is again due to increasing prevalence rates of these conditions with age alongside the projected population ageing.

**Fig. 4:**
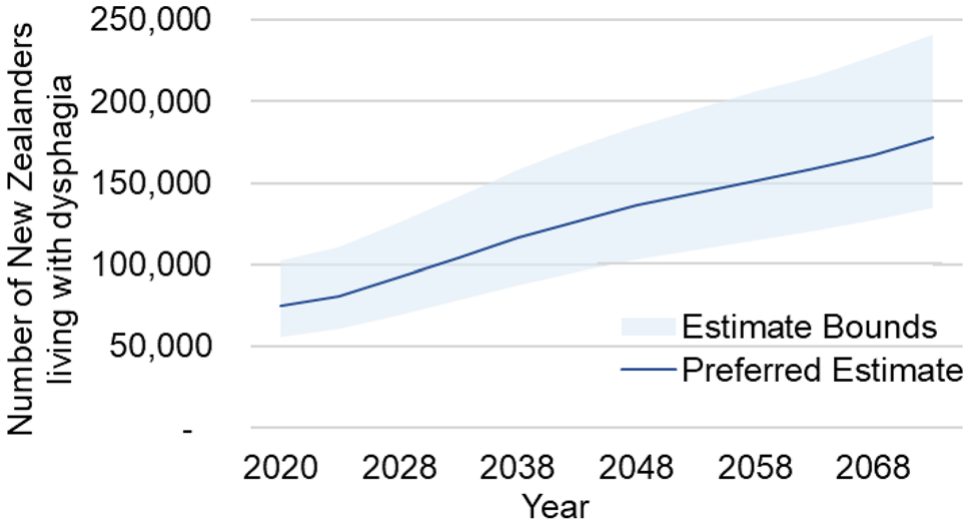
Projected Dysphagia Prevalence in New Zealand 2020–2073—Upper and Lower Bounds

Lower and upper bounds of prevalence estimates are included in Fig. 4. Given that the 2020 upper bound is below the 2073 lower bound, we can be confident in projecting an increasing dysphagia prevalence in New Zealand.

Sex-specific data represents 85% of the total prevalence estimates in 2020 and 92% in 2073. This is due to having sex-specific data only for some underlying medical conditions. Based on this sub-sample, we calculate that 51% of ongoing dysphagia cases are among men and 49% among women in both 2020 and 2073. It is therefore unlikely that there will be large sex disparities in the number of individuals living with dysphagia in the future. Age-specific data is available for 78% of the total prevalence estimates in 2020 and 87% in 2073. We calculate that 21% of patients were under 65 years and 79% were 65 + in 2020. By 2073, the proportion of dysphagic patients under 65 is predicted to decrease to 10% and those 65 + to increase to 90%. Dysphagia is therefore likely to become even more of an older-age condition in the future.

### Comparison with Existing Literature

**Fig. 5:**
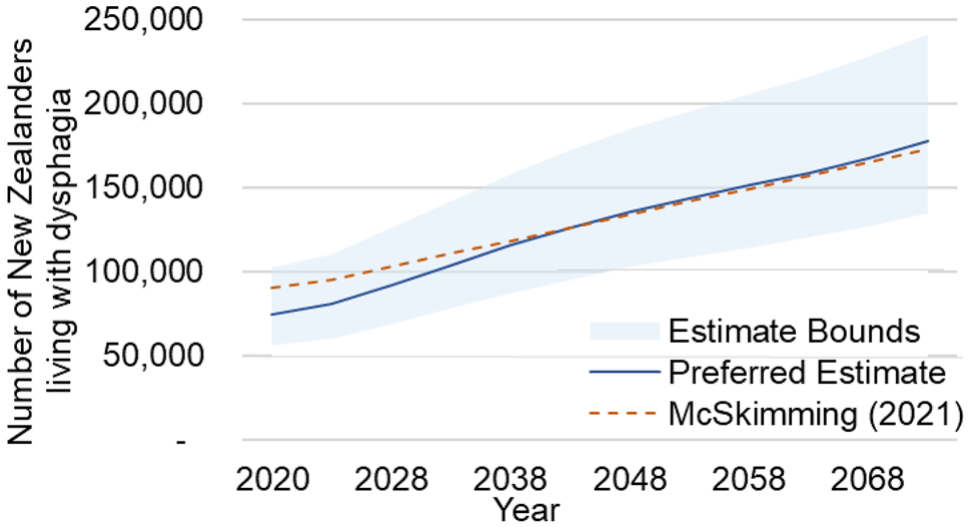
Comparing Dysphagia Prevalence Projections with McSkimming [3]

The historical prevalence of dysphagia in New Zealand was estimated using data from the Global Burden of Disease Study [3]. These estimates were then used to forecast the prevalence of dysphagia in New Zealand up to 2073. Figure 5 compares McSkimming’s [3] forecasted estimates to the projections calculated in this paper. McSkimming [3] estimates a moderately higher total prevalence in years 2020–2033, suggesting our preferred estimates may be conservative. By 2038, our estimates and McSkimming’s converge and remain largely the same until 2073. Importantly and reassuringly, McSkimming’s estimates remain within our estimate bounds over the entire projection period despite using a different dataset and estimation method.

## Conclusions and Discussion

In this paper, we draw on existing literature to calculate estimates of the incidence and prevalence of dysphagia in New Zealand. Overall, we find it is likely that both the number of newly diagnosed individuals and the total number of New Zealanders living with the effects of dysphagia will increase in the future. There is unlikely to be significant sex disparities but the already high proportion of elderly individuals newly diagnosed and living with dysphagia is likely to increase even further as the population ages. These projections provide a useful starting point to assess future healthcare resource demands associated with dysphagia, assisting with healthcare provision planning and evaluating the cost-effectiveness of new medical interventions designed to aid in the treatment or management of dysphagic patients.

Data gaps result in limitations of our analysis. For example, in the absence of demographic-specific incidence and prevalence data for certain underlying medical conditions, we are only able to show the ethnicity, age and sex composition of a portion of the total dysphagia cases.

Additionally, due to limitations in information about dysphagia onset within degenerative medical conditions such as Parkinson’s disease or dementia, we have imposed the simplifying assumption that where dysphagia arises, it arises together with the underlying condition. In reality, dysphagia is likely to be less prevalent among newly diagnosed dementia or Parkinson’s disease patients than in more advanced cases. This also implies that our confidence in the estimates of the incidence of dysphagia is higher than for the prevalence of dysphagia.

A further limitation of this work is the assumption that incidence and prevalence rates remain constant into the future. This assumes no future improvements in healthcare or other health inputs that can lead to improved health outcomes. Medical innovations that could reduce the incidence of medical conditions in the future are also assumed away in this analysis. Our work also does not provide a detailed comparison of paediatric and adult dysphagia and we note this should be the focus of separate work to gain a deeper insight into the unique implications of dysphagia within this specific cohort.

Regardless of these limitations, it can be argued that the estimates in this work are the best achievable current approximation and future projection of the incidence and prevalence of economically significant dysphagia in a New Zealand setting. By excluding relatively minor definitions of dysphagia from our analysis, we enrich the international literature by estimating the extent to which dysphagia is impacting our population.

## Data Availability

The datasets generated during and/or analysed during the current study are available from the corresponding author on reasonable request.

## Appendix

See Table 1 and 2.

**Table 1:** Summary of incidence and prevalence rate sources

Medical condition		Incidence rates: source		Prevalence rates: source	
		Ethnicity- and age-specific	Sex- and age-specific	Ethnicity- and age specific	Sex- and age-specific
Stroke		Feigin et al. [6]	Feigin et al. [6]		Bonita, Solomon and Broad [22]
Parkinson’s Disease		Pitcher et al. [7]	Myall et al. [10]	Pitcher et al. [7]	Myall et al. [10]
Dementia		Alzheimers New Zealand [8]	Public Health Agency of Canada [11]		Alzheimers New Zealand [8]
Traumatic Brain Injury		Feigin et al. [9]	Te Ao et al. [12]		Te Ao et al. [12]
Paediatric Dysphagia	Cerebral Palsy		Neurological Foundation [13]		Neurological Foundation [13]
	Craniofacial Disorder		Thompson et al. [14]		Mossey & Catilla [23]
	Autism Spectrum Disorder		Honda et al. [15]		Ministry of Health [24]
	Down's Syndrome		Ministry of Health [16] Presson et al. [17] Tidy & Bonsall [18]		Presson et al. [17] Tidy & Bonsall [18]
Cancers	Head and Neck		Ministry of Health [19]		Auckland Head and Neck Specialists [25]
	Thyroid		Ministry of Health [19]		National Cancer Institute [26]
	Oesophageal		Ministry of Health [19]		American Society of Clinical Oncology [27]
Multiple Sclerosis			Multiple Sclerosis New Zealand [20]		Multiple Sclerosis New Zealand [26]
Motor Neurone Disease			Motor Neurone Disease New Zealand [21]		Motor Neurone Disease New Zealand [28]

**Table 2:** Summary of dysphagia prevalence within medical conditions

Medical condition		Dysphagia prevalence			
		Lower Bound (%)	Upper Bound (%)	Preferred Estimate (%)	Source
Stroke		37	78	50	Martino et al. [29]
Parkinson’s Disease		72	87	80	Kalf et al. [30]
Dementia		19.6	32.8	26	Australian Institute of Health [31] Alagiakrishnan, Bhanji & Kurian [32]
Traumatic Brain Injury		2	6	4	Barker-Collo et al. [33] Field & Weiss [34]
Paediatric Dysphagia	Cerebral Palsy	33	83	58	Lefton-Greif [35]
	Craniofacial Disorder	33	83	58	Dharmarathna & Perera [36]
	Autism Spectrum Disorder	4	4	4	Ministry of Health [24] Field, Garland, & Williams [37]
	Down's Syndrome	33	83	58	Field, Garland, & Williams [37] Lefton-Greif [35]
Cancers	Pre-Treatment	28	28	28	Logemann et al. [38]
	Post-Treatment	45	51	45	Garcia-Peris et al. [39] Hutcheson et al. [40]
Multiple Sclerosis		24	81	43	De Pauw et al. [41] Guan et al. [42] Aghaz et al. [43]
Motor Neurone Disease		60	86	80	Romero-Gangonells et al. [44] Waito et al. [45]

## Footnotes

1 Statistics New Zealand project age- and sex-specific population estimates further into the future than ethnicity-specific population estimates (year 2073 vs. 2038).
